# Pharmaceutical adsorption and ex-situ electro-regeneration performance of magnetically modified activated carbon

**DOI:** 10.1007/s11356-026-37655-6

**Published:** 2026-03-25

**Authors:** Faisal Ahmed, Mohamed S. Gaber, Gamze Ersan, Sergi Garcia-Segura, Mahmut S. Ersan

**Affiliations:** 1 Department of Civil Engineering, University of North Dakota, Grand Forks, ND 58202-8115 USA; 2 School of Sustainable Engineering and The Built Environment, Arizona State University, Tempe, AZ 85287-5306 USA

**Keywords:** Adsorption, Ex-situ electro-regeneration, Iron impregnation, Carbonaceous materials, Magnetic properties, Antibiotics, Drugs

## Abstract

This study investigated the impact of iron (Fe) modification on the physicochemical and adsorption properties of powdered activated carbon (M-PAC) for pharmaceutical removal, as well as the ex-situ electro-regeneration of pharmaceutical-laden M-PAC. The objectives were to (i) examine the adsorption capacity of Fe-modified M-PAC for ciprofloxacin (CIP) and ibuprofen (IBU) in deionized water, (ii) assess adsorption capacity in wastewater effluent, and (iii) evaluate ex-situ electro-regeneration of spent M-PAC. Fe-modification improved PAC’s settling velocity by 1.7 times compared to pristine PAC, enabling easier separation without filtration or coagulation/flocculation, which benefits regeneration processes. M-PAC also exhibited more linear adsorption isotherms than pristine PAC, offering consistent and predictable adsorption behavior across varying CIP and IBU concentrations. In wastewater effluent, adsorption decreased by 28% for CIP and 67% for IBU due to stronger competition from effluent organics. Despite this reduction, Freundlich adsorption capacities of 28.7 mg/g for CIP and 15.47 mg/g for IBU were achieved. Electro-regeneration restored up to 100% of adsorption capacity within 60–180 min over a single regeneration cycle. IBU desorbed rapidly within the first hour, while CIP required longer treatment due to stronger interactions with the carbon surface. In mixed-solute systems, regeneration efficiency was reduced by approximately 25% for IBU and up to 65% for CIP, likely from competitive desorption and differing molecular affinities for M-PAC. The calculated energy consumption for electro-regeneration of spent M-PAC was < 0.2 kWh/g, making it more competitive than existing regeneration methods. Overall, the results demonstrate that Fe-modification enhances PAC’s separation and adsorption characteristics while enabling effective ex-situ electro-regeneration. Adsorbent surface properties, along with pharmaceutical molecular structure, charge, and polarity, critically influence adsorption behavior and regeneration kinetics. These findings highlight the potential of Fe-modified PAC as a reusable adsorbent for pharmaceutical removal in water treatment.

**Supplementary Information:**

The online version contains supplementary material available at 10.1007/s11356-026-37655-6.

## Introduction

With the increasing global consumption of pharmaceuticals and the extended use of over-the-counter medications, there is a growing concern about the unintended environmental impact of these metabolically active compounds. Among all pharmaceutical medicines, antibiotics and nonsteroidal anti-inflammatory drugs (NSAIDs) are the most detected pharmaceutical contaminants in the aquatic environment (Abdulrahman et al. 2025; Hama Aziz et al. 2025; Patel et al. 2016). Fluoroquinolone antibiotics such as ciprofloxacin (CIP) are effective against a broad spectrum of bacteria. Almost 90% of ingested antibiotics are usually excreted through human urine (Janecko et al. 2016; Ersan et al. 2024). Due to the high stability of antibiotics, these chemicals can resist biodegradation during wastewater (WW) treatment, leading to their potential discharge into surface waters. Prolonged exposure to low-level concentrations of antibiotics can accelerate the development of multidrug-resistant bacteria, disrupt aquatic ecosystems, and impact the health of living organisms (Thai et al. 2023; Vazquez-Roig et al. 2011). In a similar fashion, NSAID drugs such as ibuprofen (IBU) have been linked to adverse impacts on aquatic species by building up in their tissues and disrupting the endocrine systems of several species (Patel et al. 2016). The NSAIDs are extensively used for pain relief and to decrease inflammation. While the wide use of pharmaceuticals makes them almost ubiquitous pollutants in household and hospital WW effluents, their recalcitrant characters result in their detection even after WW treatment (aus der Beek et al. 2015; Shearer et al. 2022; Watkinson et al. 2009).

Among the physicochemical separation technologies, adsorption processes have been identified as the most promising and cost-effective method for antibiotic removal from WW and during the tertiary treatment in potable water reuse applications (Fanourakis et al. 2020). Granular activated carbon (GAC) and powdered activated carbon (PAC) have been frequently used as an adsorbent for the removal of various contaminants from water (Brasquet and Le Cloirec 1997; Luján-Facundo et al. 2019). Powdered activated carbon (PAC) is generally more effective than granular activated carbon (GAC) for removing pharmaceutical compounds due to its smaller particle size, higher external surface area, shorter intraparticle diffusion paths, and faster adsorption kinetics, which enhance mass transfer efficiency (Kårelid et al. 2017). However, PAC presents practical challenges in full-scale water treatment because its fine particles (10–100 µm) have slow settling velocities (0.5–1 m/h), making separation and recovery difficult. As a result, additional sedimentation or filtration steps are often required before regeneration, and the process can generate pharmaceutical-laden waste adsorbents, raising environmental sustainability concerns (Margot et al. 2013; Woermann et al. 2020; Morsch et al. 2021; Bakkaloglu et al. 2021; Krahnstöver and Wintgens 2018).

However, the recovery and regeneration of PAC after its use is challenging and not environmentally sustainable due to the small size particle of PAC (10–100 µm) with slow settling velocities (0.5–1 m/h) and generation of pharmaceutical-laden waste adsorbents (Bakkaloglu et al. 2021; Krahnstöver and Wintgens 2018). Therefore, water treatment plants implement the use of coagulants to improve the settling properties of PAC (40–50 m/h) from the treated water and seeking cost-effective and environmentally sustainable regeneration approaches. Engineered PAC through magnetic functionalization can increase the settling performance and its recovery after treatment, which would avoid the need for additional post-treatment steps that generate sludge.

Various ex-situ regeneration methods have been used to regenerate exhausted activated carbons, including thermal regeneration (Márquez et al. 2022), solvent regeneration (Siriwardena et al. 2021), and microwave regeneration (Gagliano et al. 2021). Although these conventional ex-situ regeneration techniques can effectively regenerate spent activated carbons, they are not environmentally friendly, cost-effective ($2.0–2.9/kg) (Ling et al. 2025), and highly energy-intensive (> 900 kWh/kg) (Gagliano et al. 2021; Ersan et al. 2023c), and they may generate hazardous byproducts. One promising ex-situ strategy is to couple adsorption with electrification to enable electro-regeneration of the adsorbent. Electro-regeneration is particularly appealing for spent activated carbons due to its conductive properties and low energy requirement (< 0.9 kWh/g) (Ersan et al. 2023c). Electro-regeneration technology relies on anodic/cathodic polarization. When a cathodic current is applied to exhausted activated carbons, it becomes negatively charged, leading to desorption of the adsorbed contaminants into the bulk solution, moving toward the oppositely charged anode. Under these electrified conditions, the released contaminants may also undergo electrochemical degradation due to their oxidation or reduction. Nevertheless, electro-regeneration of pharmaceutical-laden activated carbon remains insufficiently studied, particularly when the carbon surface has been modified.

Incorporation of metal species in activated carbons (AC) can also alter surface charge, enhance electrostatic interactions and chemical bonding with organic pollutants, increase the density of particles which may result in faster settling rates, and increase electrical conductance for electro-regeneration (Zhou et al. 2019; Ersan et al. 2023a, 2024; Li et al. 2024). Recent studies have demonstrated that impregnating metals such as Ag and Fe onto GAC enhanced the selectivity of adsorbents for CIP uptake in urine matrices (Ersan et al. 2023b) while also improving adsorbents’ conductivity for electro-regeneration (Ersan et al. 2024). To date, most studies have focused on the metal modification of GAC and AC adsorbents. While only a limited number of studies have investigated the modification of PAC (Jafari et al. 2016; Jothinathan et al. 2022) for pharmaceuticals adsorption in different background matrices, the sequential electro-regeneration of pharmaceutical-laden M-PAC has not been investigated. If successfully synthesized, magnetically enabled PAC (M-PAC) can become a game changer in terms of ease of recovery and regeneration without the need of chemical addition.

In the present study, the adsorption of one common antibiotic (CIP) and one NSAID drug (IBU) on M-PAC was investigated in both single-solute (deionized water (DI)) and multisolute (WW) backgrounds, along with the electro-regeneration of pharmaceutical-laden M-PAC. The main objectives of this study are to (i) assess the physicochemical properties and adsorption capacity of Fe-modified M-PAC on the settling and adsorption efficiency of two selected pharmaceuticals, ciprofloxacin (CIP) and ibuprofen (IBU), in single-solute background (deionized water); (ii) investigate the effect of background water on the adsorption of both CIP and IBU on M-PAC; and (iii) evaluate the electro-regeneration of CIP- and/or IBU-laden M-PAC.

## Materials and methods

### Materials

CIP and IBU were purchased from Sigma-Aldrich (CAS: 85721–33-1, ≥ 98% and CAS: 15687–27-1, ≥ 98%, respectively). The physicochemical properties of CIP and IBU are presented in Table S1 in the supporting information (SI) section. The phosphoric acid (H_3_PO_3_, CAS: 7664382, < 85%), acetonitrile (CH_3_CN, HPLC Grade, ≥ 99.9%), and ethanol (C_2_H_5_OH, CAS: 64175) were purchased from Sigma-Aldrich and used for the analysis of pharmaceuticals. Magnesium sulfate (MgSO₄, ≥ 99%, CAS: 7487–88-9), sodium hydroxide (NaOH, ≥ 97%, CAS: 1310–73-2), and hydrochloric acid (HCl, 36%, CAS: 7647–01-0) were obtained from Fisher Scientific. Sodium chloride (NaCl, 99%, CAS: 7647–14-5) was acquired from Acros Organics, and nitric acid (HNO₃, CAS: 7697–37-2) was purchased from LabChem. CIP and IBU stock solutions were prepared in DI water. In this study, the pH of the solutions was adjusted to pH 7.0, using 10 mM phosphate buffer solution. The sodium dihydrogen phosphate monohydrate (NaH_2_PO_4_.H_2_O, 99%) and sodium phosphate dibasic heptahydrate (Na_2_HPO_4_.7H_2_O, 99%) were purchased from Sigma-Aldrich and used for preparing the phosphate buffer.

The secondary-treated WW effluent sample was collected from a local conventional WW treatment plant in North Dakota (ND), filtered through 0.45 µm filter, and stored at 4 °C in the lab. Prior to use, the WW effluent sample was brought to room temperature (25 ± 2 °C). The characterization of the WW effluent water is presented in Table 1.

**Table 1.** Characteristics of treated WW effluent

Parameters	Value(s)
pH	8.15
Conductivity (µS/m)	1556
Ammonia (mg/L as N)	0.12
Nitrate (mg/L as N)	2.8
Phosphate (mg/L)	3.8
Dissolved organic carbon (mg/L)	12.4
Dissolved organic nitrogen (mg/L as N)	25.7
Total nitrogen (mg/L as N)	28.6
SUVA_254_ (L/mg-m, calculated as UV_254nm_/DOC)	2.2

*SUVA*_*254*_ specific UV absorption rate at 254 nm

### Synthesis and characterization of M-PAC

Coal-based GAC (Filtrasorb 400 from Calgon Corp.) was used to prepare PAC and synthesize the magnetic Fe-doped PAC or M-PAC. A coffee grinder was used to grind GAC into PAC, then ground PAC was wet sieved through 170 US mesh size (~ 88 µm) and dried in the freeze dryer for 24 h (Harvest Right Freeze Dryer). Later, the M-PAC was prepared according to previous literature with some modifications (Oliveira et al. 2002). In brief, 28 mM FeCl_3_·6H_2_O and 14 mM FeSO_4_·7H_2_O were dissolved in 400 mL of DI water. The amount of PAC was adjusted to obtain PAC/iron oxide weight ratios of 1:1. The produced iron oxide solution was stirred with pristine PAC at 700 rpm. The suspension was heated to a temperature range of 70–80 °C. Later, 5.0 M NaOH solution was gradually added dropwise into the suspension solution until the pH reached a range of 10–11. The hydrated iron oxide was precipitated from the solution during this step. After 1 h, the suspension was cooled down to room temperature. Then, the Fe_3_O_4_-PAC composites were separated from the suspension using a neodymium magnet. The separated material was subsequently washed with DI water and pure ethanol until the solution pH became neutral. Any residual reactants or byproducts from the surface of the adsorbents were removed during this thorough rinsing process. After several rinsings, M-PAC was dried in the freeze dryer.

The Brunauer–Emmett–Teller (BET) surface areas and pore size distributions of M-PAC were measured by a Tristar II 3020 analyzer at 77 K. To determine the surface elemental contents of M-PAC, surface elemental analysis was performed using a Kratos X-ray photoelectron spectroscopy (XPS) instrument. The Fe contents of M-PAC were measured in the solution of digested samples using inductively coupled plasma mass spectroscopy (ICP-MS) analysis (Thermo Scientific Quadrupole ICP-MS). For acid digestion, 25 mL nitric acid (HNO_3_, 68%) was added to a 170 mg carbon sample. Then, the mixture was boiled (150 °C) for 2 h. During the acid digestion, the inner walls of the PTFE beakers were washed with 2 mL of DI water to prevent the loss of the sample (Gaudino et al. 2007; Uddin et al. 2016). Later, all the samples were filtered and diluted with ultrapure water to 10 mL and then analyzed with ICP-MS. The point of zero charge (pH_pzc_) was measured using a modified method of Stumm and Morgan (Stumm and Morgan 1996). Briefly, a 0.1 M NaCl solution was boiled, cooled to room temperature, and transferred into 50 mL test tubes. The initial pH of the samples was adjusted from pH 3 to 12, and 25 mg of the M-PAC was transferred into the test tubes. The test tubes were placed on an orbital shaker for 24 h, after which the final pH in the test tubes was measured. The pH_pzc_ is obtained from pH_final-initial_ versus pH_initial_ plot, where the line intersects the zero line.

### Adsorption isotherm experiments and analytical techniques

Single point constant dose batch adsorption isotherm experiments for both CIP and IBU were conducted using 40 mL amber bottles with Teflon lined screw caps at room temperature (25 ± 2 °C). One milligram of M-PAC was transferred into the 40 mL bottles. The experiments were performed in DI water. The bottles were filled head space free with the DI water and then spiked with predetermined trace concentrations of CIP or IBU (ranging from 0.5 to 5.0 mg/L) from the stock solution (50 mg/L). After that, each isotherm bottle was placed on a shaker table for 72 h at 250 rpm. The equilibrium time of each isotherm bottle was selected as 72 h for all adsorbents because our initial kinetic experiments showed this was long enough to attain equilibrium. A longer contact time was employed to reach equilibrium for all tested concentrations and to account for potential slow intraparticle diffusion into microporous regions of the adsorbent. To track the loss of adsorbates during the experiments, which was shown to be minimal, bottles without adsorbent were used as “blanks.”

The duplicate analysis of CIP and IBU was performed for selected samples using the High-Performance Liquid Chromatography (HPLC-Agilent Technologies, USA). The HPLC system was equipped with an Agilent Eclipse Plus C18 column (particle size 3.5 µm, 4.6 mm × 100 mm) and a DAD detector. The detection limit for CIP and IBU was 0.1 mg/L. The analytes were isocratically eluted with a mixture of acetonitrile (40%) and 0.1% phosphoric acid (60%). The column was maintained at 30 °C with a flow rate of 1.0 mL/min. The injection volume was 50 μL for CIP and 40 μL for IBU. The standard deviation for the samples was within ± 5%.

Two common adsorption isotherm models, Freundlich and Langmuir, were employed to analyze the data and determine the best fit. The coefficient of determination (*R*^2^) values and isotherm model parameters for both models were calculated, and all the equations are presented in Table S2.

### Ex-situ electro-regeneration of spent M-PAC

**Fig. 1:**
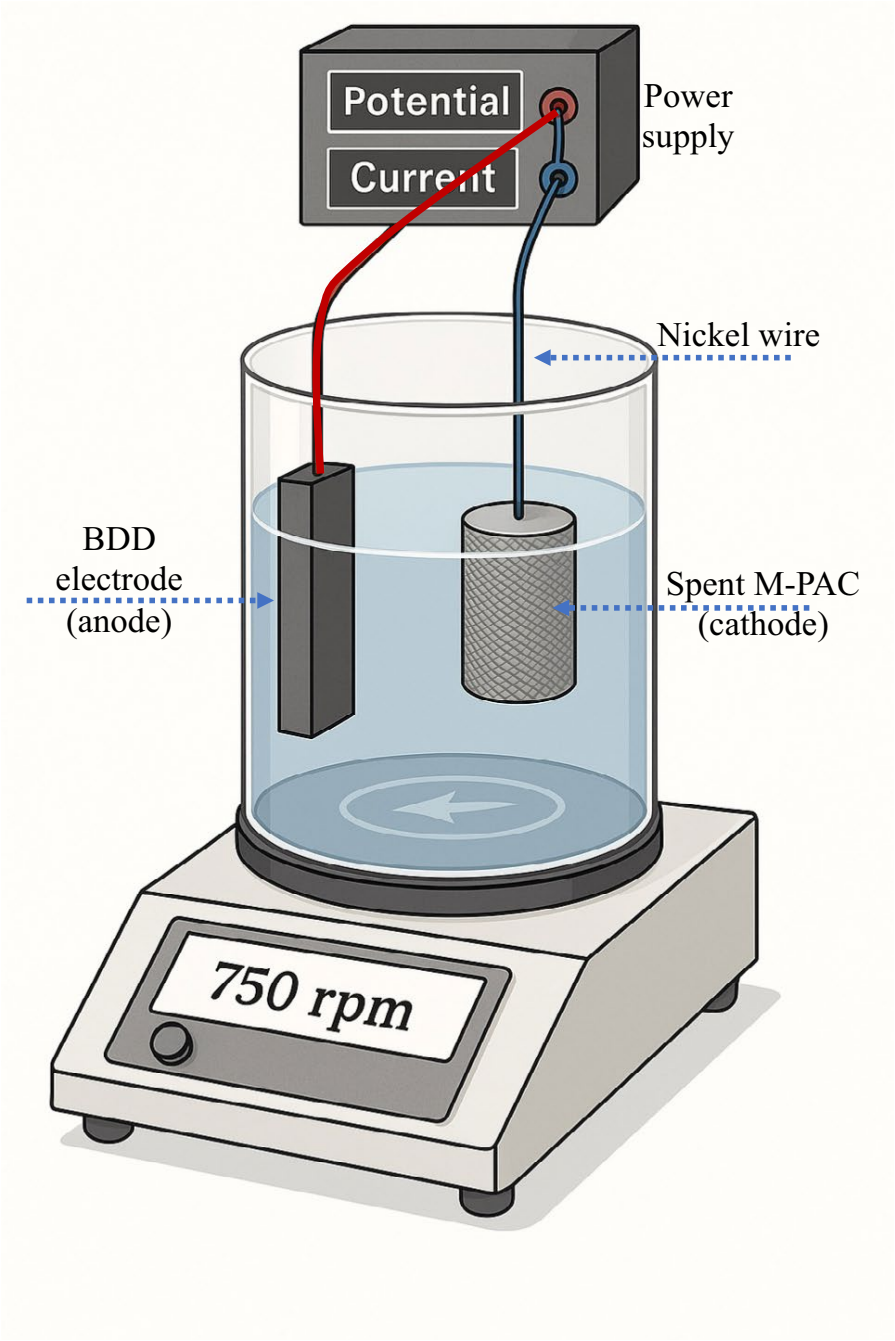
Electro-regeneration batch reactor set-up

Ex-situ electro-regeneration batch reactor experiments (Fig. 1) were conducted using a 200 mL cylindrical electrochemical cell containing 150 mL of 250 mM Na₂SO₄ solution as the supporting electrolyte. The electrolyte solution was continuously mixed using magnetic stirring at 750 rpm to ensure homogeneous mixing in the cell. A NP6005 power supply was used to carry out galvanostatic experiments, with a cell potential of 30 V and a constant current density of 60 mA/cm^2^. A boron-doped diamond (BDD) electrode with an active surface area of 3 cm^2^ was employed as the anode, maintaining an interelectrode gap of 1 cm between the anode (BDD) and the cathode (spent M-PAC). The BDD thin films, provided by NeoCoat SA (Switzerland), were synthesized on p-doped monocrystalline Si (100 mΩ·cm) with 2500 ppm of boron doping. The reactor compartments contained a concentration of 100 mg/L of CIP, IBU- or CIP/IBU-loaded M-PAC, along with 100 mg of spent M-PAC. The pharmaceutical-laden M-PAC was transferred into a stainless steel mesh and placed in the cathode compartment. To evaluate the regeneration of spent M-PAC loaded with CIP and IBU during the electro-regeneration experiment, samples were collected at different time intervals and analyzed using HPLC, as described in the previous section. CIP was extracted prior to HPLC analysis to eliminate salt interference that could affect the detection accuracy in HPLC analysis. The additional extraction step was necessary only for CIP due to its zwitterionic nature and strong interactions with sulfate ions in the supporting electrolyte, which can cause chromatographic interference during HPLC analysis, whereas IBU, being predominantly neutral and more hydrophobic under the experimental conditions, did not exhibit noticeable salt interference and was analyzed directly. The extraction of CIP was performed using the salting-out assisted liquid–liquid extraction method described in the literature (Gezahegn et al. 2019). Briefly, 5 mL of the CIP or mixed CIP/IBU-containing sample was transferred into a 10 mL screw-capped polyethylene tube. Then, the sample pH was adjusted to 3 using 1 N HCl and NaOH to convert CIP to its neutral form, enhancing extraction efficiency. Subsequently, 2.5 mL of acetonitrile (CH_₃_CN) was added, and the mixture was shaken vigorously for 1 min. Thereafter, 2.0 g of anhydrous magnesium sulfate (MgSO_₄_) was introduced to promote phase separation. The solution was then stirred for 6 min to ensure complete dissolution of the salt. Following that, the mixture was centrifuged for 5 min at 4000 rpm. Using a microsyringe, the upper acetonitrile organic phase was carefully collected and the extract was then dried at 85 °C in a water bath. Finally, the dried extract was reconstituted with 1 mL of distilled water before being transferred to a 2 mL sample vial for analysis with HPLC system. Electro-regeneration efficiency and capacity were calculated using Eqs. (1) and (2) (Le et al. 2024; Gaber et al. 2023). Additionally, the electrical energy required to regenerate spent M-PAC was calculated using the energy consumption (EC, kWh/g) values based on Eq. (3) (Ersan et al. 2023c).

1 $$RE=\left(\%\right)=\frac{C_t}{C_o}\times100$$

2 $$\mathrm{RC}\;(q{\scriptsize{t}})(mg/g)=\frac{\mathrm{Ct}\times{\mathrm{V}}}{\mathrm{M}}$$

3 $$EC=\frac{Ecell\times I\times t}{1000\times M-PAC\;mass}$$

where RE is regeneration efficiency, *C*_t_ is the concentration (mg/L) of CIP or IBU after a proper time, *C*_o_ (mg/L) is the initial concentration of CIP/IBU, RC is regeneration capacity (mg/g), *V* is the volume of the solution (L), *M* is the mass of the adsorbent used (g), *E*_cell_ is the potential differences through the electro-regeneration system (V), *I* is the applied current (A), *t* is the charging time (h), M-PAC_mass_ is the mass of exhausted M-PAC (g), and 1000 is a conversion factor.

## Results and discussion

### Characterization of magnetically enabled adsorbent

The physicochemical properties of GAC, PAC, and M-PAC were characterized and are presented in Table 2. The distinctions in their physicochemical properties were evaluated by considering their surface area, pore volume, pore size distribution, surface functional groups, surface charge, and thermogravimetric composition. Overall, while GAC and PAC exhibited similar BET surface area (1005 m^2^/g and 1039 m^2^/g, respectively) and total pore volume of (0.49 cm^3^/g and 0.56 cm^3^/g, respectively), the BET surface area (562 m^2^/g) and total pore volume (0.33 cm^3^/g) of M-PAC were ~1.8-fold lower than that of its pristine counterpart (PAC). The decrease can be ascribed to pore blockage by Fe-modification on the PAC surface. This is supported by the decrease in the total pore volume of PAC from 0.557 to 0.331 cm^3^/g after Fe-modification. M-PAC predominantly contained micropores, which are known to offer plentiful high-energy sites for adsorption of CIP and IBU. The pH_pzc_ values of GAC, PAC, and M-PAC were measured and are presented in Table 2. The results indicated that while GAC and PAC had similar pH_pzc_ (9.5 and 10.0, respectively), M-PAC had a pH_pzc_ of 6.6. This drastic change on pH_pzc_ suggests that while the surface of PAC was positively charged under experimental conditions (DI water pH 7.0 and WW pH 8.15), the M-PAC was nearly neutral (at pH:7) and negatively charged (at pH 8.15), respectively. This change on surface charge is associated with the increase of oxygen functional groups on the carbon surface (Dittmann et al. 2022).

**Table 2:** Physicochemical properties of GAC, PAC, and M-PAC

PAC	SSA_BET_^a^	*V*_T_^b^	*V*_T_^c^		Surface elemental analysis (%)			pH_pzc_
			***V***_**micro**_	***V***_**meso/macro**_	**Carbon**	**Oxygen**	**Iron**	
	**(m**^**2**^**/g)**	**(cm**^**3**^**/g)**	** < 2 nm**	** > 2 nm**				
**GAC**	1005	0.49	0.32	0.141	92.7	7.3	N.D.^d^	9.5
**Pristine PAC**	1039	0.56	0.31	0.25	92.6	7.4	N.D.^d^	10
**M-PAC**	562	0.33	0.17	0.16	23.7	31.0	45.3	6.6

^a^SSA_BET_: specific surface area

^b^*V*_T_: total pore volume

^c^The pore volume fractions in the micro-, meso-, and macropore size ranges were determined using density functional theory (DFT) analysis from BET measurements

^d^N.D.: not detected

**Fig. 2:**
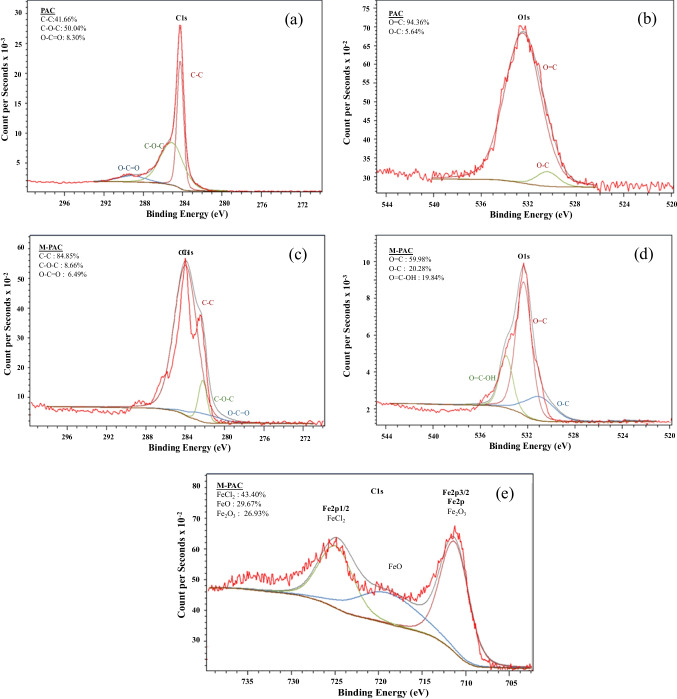
The deconvolution of (**a**) C 1 s, (**b**) O 1 s for PAC and (**c**) C 1 s, (**d**) O 1 s, and (**e**) Fe 2p_1/2_ and Fe 2p_3/2_ for M-PAC

Figure 2a–e shows the XPS results of both pristine and M-PAC, which wide spectra of M-PAC revealed peaks associated with carbon, oxygen, and iron. The XPS analyses of PAC (Fig. 2a, b) and M-PAC (Fig. 2c–e) illustrate the shift of characteristic peaks toward higher binding energies suggesting the existence of carbon atoms that are chemically bonded to oxygen atom(s). The C–C, C–O–C, and O–C = O functional groups are present on both PAC and M-PAC surfaces (Fig. 2a, c). The C–C, C–O–C, and O–C = O functional groups carry negative charges and, especially in the case of carboxylic acids, demonstrate a strong attraction for Fe species (Chen et al. 2017; Ersan et al. 2023a). After the magnetization process (Fig. 2b versus Fig. 2d), the O = C functional groups on PAC (94.36%) drastically decreased in M-PAC by ca. half (59.98%), while the O-C functional groups on PAC (5.64%) increased in M-PAC relatively by fourfold (20.28%). The presence of the O = C–OH group on M-PAC can be attributed to the alteration of surface functional groups on the adsorbent surface (Fig. 2d). For M-PAC, the binding energies of Fe 2p1/2 and Fe 2p3/2 on XPS spectra were identified as 724.2 eV and 710.9 eV, respectively (Fig. 2e). The analysis revealed the presence of FeCl_2_ (43.40%), FeO (29.67%), and Fe_2_O_3_ (26.93%).

**Fig. 3:**
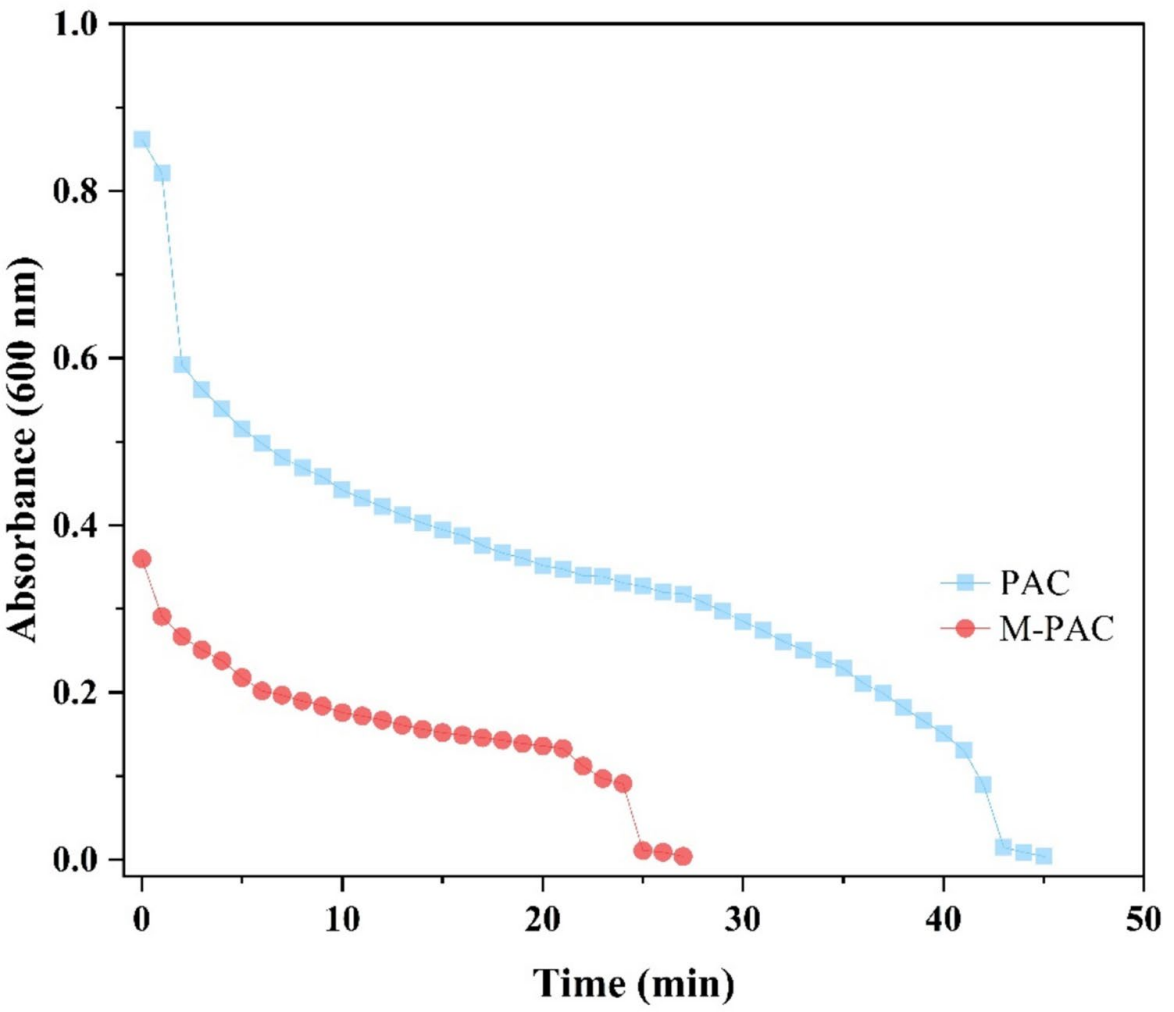
Settling test for pristine PAC and M-PAC adsorbents

The settling test for both PAC and M-PAC is demonstrated in Fig. 3. The absorbance measurements at *λ* = 600 nm were recorded for suspensions prepared at an adsorbent concentration of 1.0 g/L (3.0 mg in 3 mL of DI water) over settling times ranging from 0 to 50 min. The results showed that M-PAC exhibited two-fold faster-settling capabilities than PAC. The absorbance of M-PAC particles decreased to almost zero (0.004 a.u) within 27 min (Table S3b). In contrast, PAC took longer to settle completely, as evidenced by its absorbance of 0.004 a.u at 45 min, and its absorbance of 0.318 at 27 min as compared to M-PAC (Table S3a). This suggests that PAC with functional magnetic properties may offer benefits not only as adsorption sites for removing contaminants but also for accelerating settling in water and WW treatment processes when ex-situ regeneration is considered for sustainable reuse of this adsorbent.

### Adsorption mechanism of CIP and IBU on M-PAC in single solute system

**Fig. 4:**
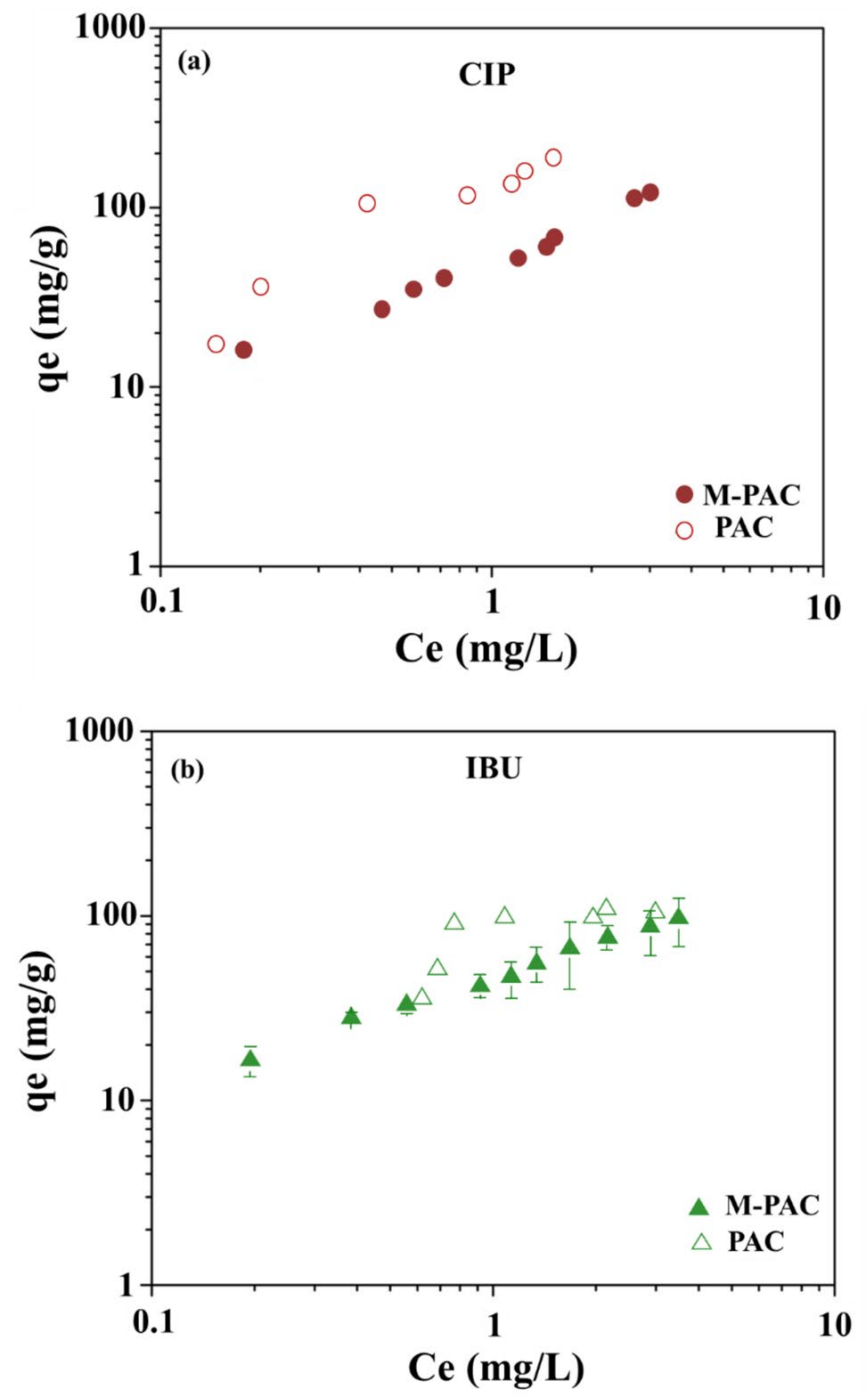
Adsorption isotherms of (**a**) CIP and (**b**) IBU on M-PAC and pristine PAC in DI water on a mass basis

Figure 4 demonstrates the adsorption isotherms of CIP and IBU on both M-PAC (Fig. 4a) and pristine PAC (Fig. 4b) and M-PAC in a single-solute DI water background. For both CIP and IBU adsorption, M-PAC exhibited a more linear adsorption isotherm trend, indicating a more consistent and predictable adsorption behavior across a wide range of CIP and IBU concentrations. In contrast, the isotherms for pristine PAC showed a stepwise pattern, suggesting a multi-stage adsorption process (initially filling high-affinity micropores, followed by adsorption on meso- and macropores with lower affinity). These results imply that M-PAC enables more controlled and uniform adsorption of CIP and IBU, whereas pristine PAC may exhibit non-linear uptake due to more heterogeneous pore filling. Despite PAC having twice the surface area of M-PAC (as shown in Table 2), their adsorption capacities are in almost similar ranges (10–110 mg/g) as demonstrated in Fig. 4. This can be ascribed to the presence of oxygen and Fe functional groups in the form of FeO, FeCl_2_, and Fe_2_O_3_ on M-PAC (Fig. 2), which could enhance specific interactions with CIP and IBU, such as hydrogen bonding or π-π interactions (Ersan et al. 2024). Additionally, the presence of residual Fe species on M-PAC may contribute to variations in adsorption behavior of CIP and IBU in pure DI background. When Fe was impregnated onto the PAC surface, the surface oxygen groups significantly increased, which led to lower pH_pzc_ (6.6) as compared to the PAC (pH_pzc_:10) (Table 2). While Fe impregnation imparted magnetic properties to the adsorbent, the adsorption of CIP on M-PAC (Fig. 4a) was slightly lower than PAC (Fig. 4b) (with positively charged surface) with highest equilibrium concentration (*C*_e_) due to the nearly neutral surface of M-PAC at the experimental pH of 7. This suggests that the adsorption mechanism was primarily governed by electrostatic interactions between the positively charged both PAC surfaces and the negatively charged carboxylic groups of CIP under the experimental pH conditions.

With increasing concentration, PAC shows a much steeper CIP uptake compared to M-PAC, which may be attributed to the microporous structure of PAC that contains high-energy sites for adsorption. On the other hand, IBU uptake was steeper until the 0.79 mg/L equilibrium concentration, then plateaued with increasing *C*_e_ concentrations on PAC. This trend suggests that the micropore filling in the microporous structure of PAC allows the relatively small IBU molecules (10.3 × 5.2 × 4.3 Å) to diffuse efficiently into the micropore regions (~ 20 Å), leading to slow saturation of available adsorption sites. In contrast, M-PAC, with its modified surface characteristics, exhibited a more gradual uptake pattern, likely due to near evenly distributed micro and meso/macro pores and neutral surface chemistry that controlled the adsorption kinetics of either large or small molecules (CIP or IBU). The physicochemical interactions between pharmaceuticals and M-PAC are expected to be more pronounced, as its surface is nearly neutral, which minimizes electrostatic repulsion and enhances the non-polar interactions. Characterization results further support this observation, indicating that the adsorption mechanism of CIP and IBU on M-PAC may be governed by a combination of hydrophobic interactions and pore accessibility.

The adsorption isotherm model (Freundlich and Langmuir) parameters and the coefficient of determination (*R*^2^) values for M-PAC were calculated and presented in Table 3. The *R*^2^ values in the Freundlich and Langmuir models for CIP adsorption on M-PAC (*R*^2^_FM and LM_ = 0.96) indicate that adsorption is characterized by mono and multi-layer adsorption on M-PAC. On the other side, the Freundlich model for M-PAC for IBU exhibited higher *R*^2^ values (*R*^2^_FM_ = 0.99) compared to the Langmuir model (*R*^2^_LM_ = 0.96). This may be attributed to the smaller size of IBU, which facilitated stronger interactions and, consequently, multilayer stacking of the molecule on the M-PAC. This finding is also supported by the slightly higher *K*_F_ values for IBU (47.7) compared to CIP (40.1). Overall, the similar adsorption capacities of CIP and IBU on M-PAC suggest that the accessibility of both contaminants to the inner and outer regions of the M-PACs was not influenced by the molecular size of the compounds.

**Table 3:** Adsorption isotherm parameters for the adsorption of CIP and IBU on M-PAC in single solute background

Ciprofloxacin (CIP)				Ibuprofen (IBU)			
**Langmuir**		**Freundlich**		**Langmuir**		**Freundlich**	
***q***_**m**_	82	***K***_**F**_	40.1	***q***_**m**_	104	***K***_**F**_	47.7
***K***_**L**_	1.15	***n***	1.69	***K***_**L**_	0.89	***n***	1.39
***R***^**2**^	0.96	***R***^**2**^	0.96	***R***^**2**^	0.96	***R***^**2**^	0.99
***N***	10	***N***	10	***N***	9	***N***	9

**Fig. 5:**
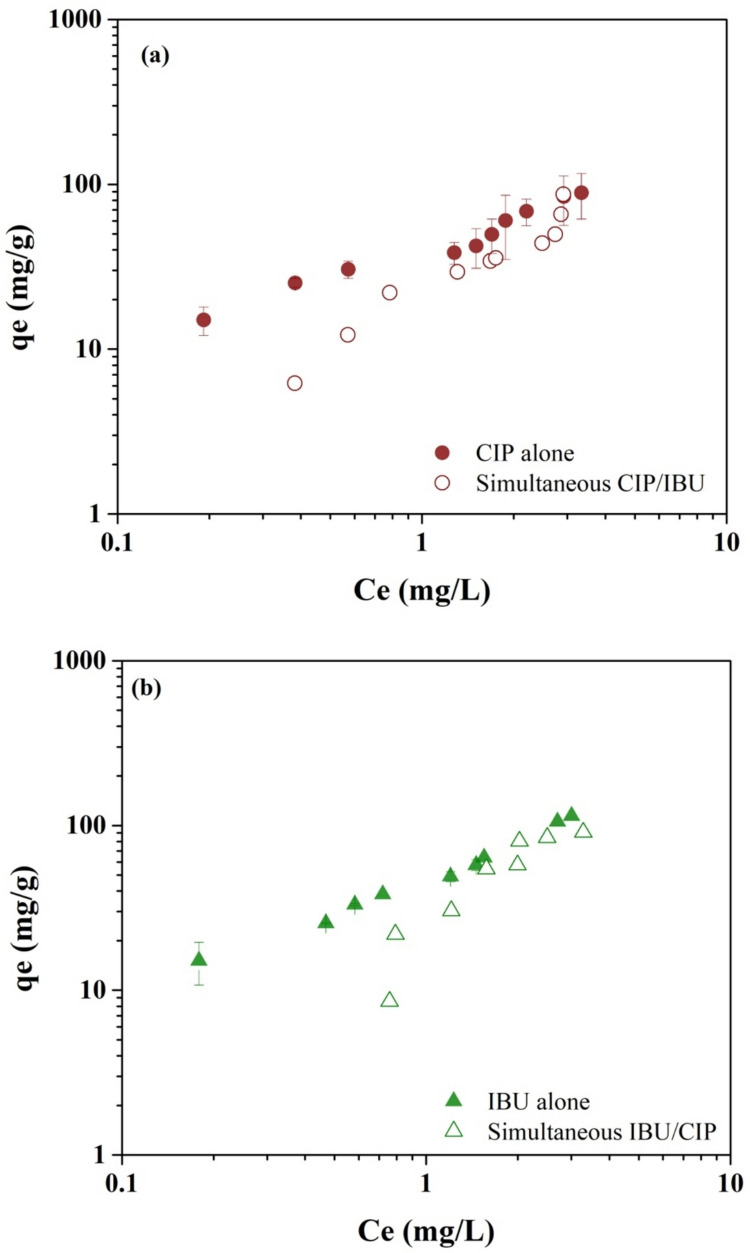
Simultaneous competitive adsorption of CIP (**a**) and IBU (**b**) on M-PAC in DI water background

In natural and engineered water systems, organic contaminants coexist, and therefore, they may compete for adsorption sites on the adsorbents. To mechanistically understand the competition between CIP and IBU molecules, simultaneous adsorption experiments were conducted for CIP (Fig. 5a) and IBU (Fig. 5b) on M-PAC in single-solute DI background.

The results showed that, during simultaneous adsorption, the adsorption of CIP decreased due to the higher competition from the hydrophobic IBU molecule (log*K*_ow_ = 3.5) against the relatively hydrophilic CIP (log*K*_ow_ = 0.42) for the near-neutral M-PAC surface. As the concentration of CIP and IBU increased (particularly IBU), the competition between CIP and IBU decreased. This suggests that the adsorption efficiency of M-PAC is influenced by the concentration of coexisting pharmaceuticals at lower equilibrium concentrations.

### Adsorption mechanism of CIP and IBU on M-PAC in multisolute system

**Fig. 6:**
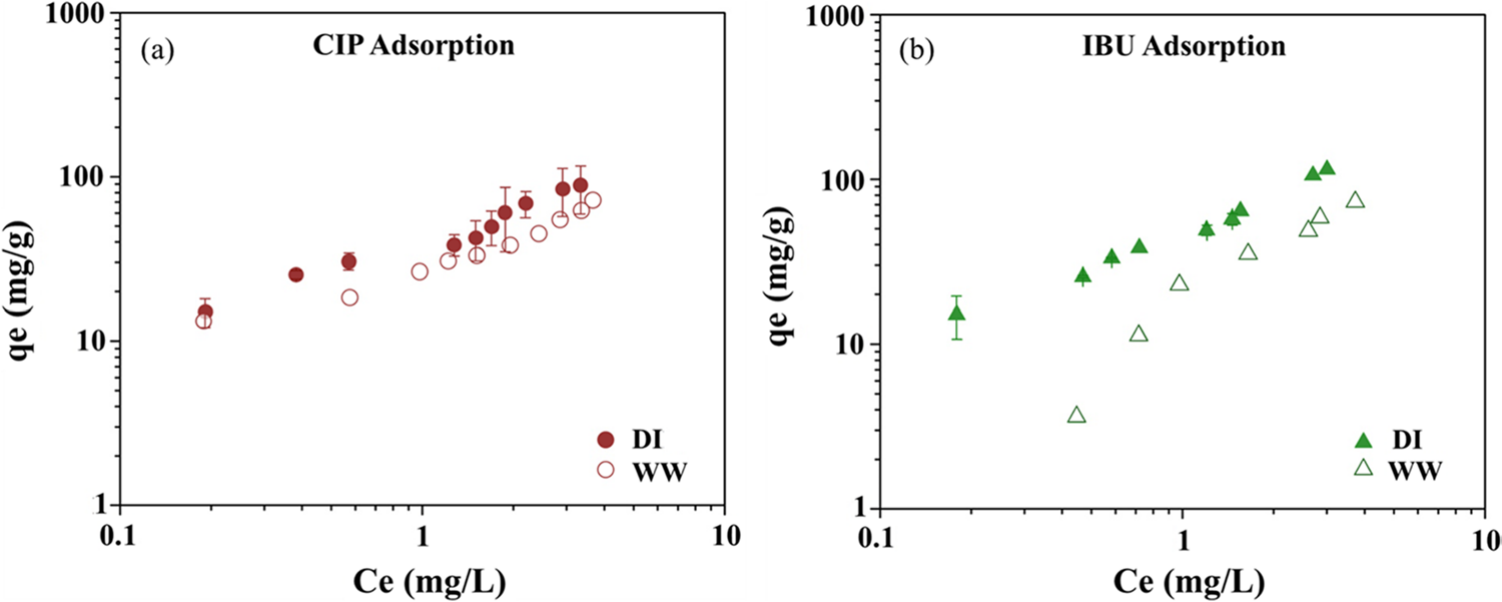
CIP (**a**) and IBU (**b**) adsorption on M-PAC in DI versus WW backgrounds

The adsorption experiments were conducted in a real WW matrix to evaluate the influence of a real water matrix on the adsorption capacities of M-PAC, compared to a DI water background. The results are shown in Fig. 6. In WW background, the adsorption capacity of both CIP and IBU was decreased for both adsorbents relative to DI water background. Specifically, the Freundlich adsorption capacity (*K*_F_) for CIP decreased from 40.1 in DI water to 28.7 in WW effluent, corresponding to an approximately 28% reduction in adsorption capacity. In contrast, IBU exhibited a more pronounced decrease, with *K*_F_ decreasing from 47.7 to 15.47, representing an approximately 67% reduction. This reduction in adsorption performance is due to the competition and pore blockage by organics in the effluent water (Ding et al. 2008; Almanassra et al. 2020), which is mainly associated with the presence of high concentration of total organic carbon and dissolved organic nitrogen (Parniske et al. 2024; Guillossou et al. 2020). Effluent organic matter in WW may contain organic molecules of both hydrophobic and hydrophilic origin with a negative charge (Shon et al. 2007). However, the net charge balance depends on the presence of other anions and cations, which may have a charge-neutralizing effect. Therefore, the decrease in the adsorption capacity of M-PAC for CIP and IBU can be attributed to pore blockage of the adsorbents by the organics, surface charge alterations by the ions, or the presence of other organic pollutants in the WW background.

The Freundlich and Langmuir adsorption isotherm model parameters and their *R*^2^ values were reported for the DI water and WW background in Table 4. In DI water background, the *R*^2^ values for Freundlich and Langmuir isotherm models exhibited similar *R*^2^ values (0.96–0.99), indicating the CIP and IBU adsorption is favored in both mono- and multilayer adsorption in pure water background. However, the *R*^2^ values for the Freundlich model describing CIP adsorption on M-PAC (*R*^2^_FM_ = 0.95) were higher than that of the Langmuir model (*R*^2^_LM_ = 0.86) in WW background. For IBU, the Freundlich model for M-PAC (*R*^2^_FM_ = 0.94) also showed a better fit than its Langmuir model (*R*^2^_LM_ = 0.88), indicating that adsorption on M-PAC surface is characterized by multilayer adsorption in the presence of WW effluent water. Overall, both pharmaceutical chemicals exhibited a good fit with the Freundlich model in the presence of WW. The Freundlich adsorption capacities of CIP on M-PAC (*K*_F_ = 28.7) were higher than that of IBU (*K*_F_ = 15.47), even though the log*K*_ow_ of IBU (3.5) is higher than that of CIP (0.42). Therefore, hydrophobic-hydrophobic interactions between IBU and the M-PAC surface are not the predominant mechanism in WW background.

**Table 4:** Effect of background water on Freundlich isotherm parameters for the adsorption of CIP and IBU on M-PAC

DI water								WW effluent							
**Ciprofloxacin**				**Ibuprofen**				**Ciprofloxacin**				**Ibuprofen**			
**Langmuir**		**Freundlich**		**Langmuir**		**Freundlich**		**Langmuir**		**Freundlich**		**Langmuir**		**Freundlich**	
***q***_**m**_	82	***K***_**F**_	40.1	***q***_**m**_	104	***K***_**F**_	47.7	***q***_**m**_	51.81	***K***_**F**_	28.7	***q***_**m**_	24.21	***K***_**F**_	15.47
***K***_**L**_	1.15	***n***	1.69	***K***_**L**_	0.89	***n***	1.39	***K***_**L**_	1.63	***n***	1.74	***K***_**L**_	0.33	***n***	0.77
***R***^**2**^	0.96	***R***^**2**^	0.96	***R***^**2**^	0.96	***R***^**2**^	0.99	***R***^**2**^	0.86	***R***^**2**^	0.95	***R***^**2**^	0.88	***R***^**2**^	0.94
***N***	10	***N***	10	***N***	9	***N***	9	***N***	10	***N***	10	***N***	7	***N***	7

### Ex-situ electro-regeneration of spent M-PAC

**Fig. 7:**
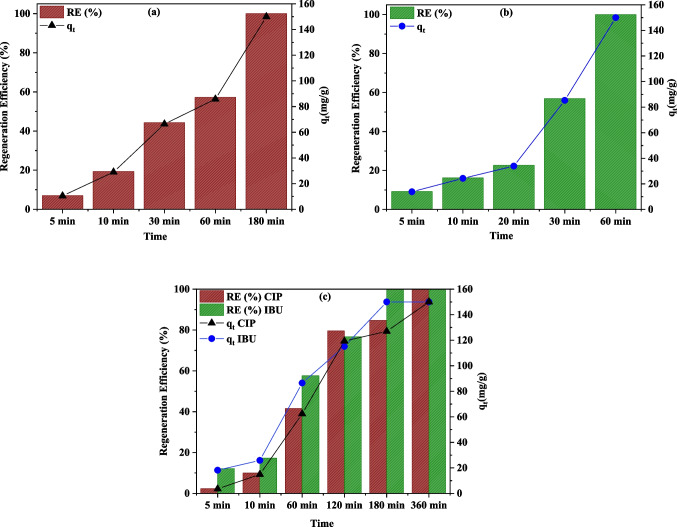
Regeneration efficiency and capacity of spent M-PAC as a function of time for (**a**) CIP alone, (**b**) IBU alone, and (**c**) CIP and IBU mix

Figure 7 illustrates the ex-situ regeneration performance of spent M-PAC loaded with CIP and IBU over time in single-solute (only CIP or IBU) and multi-solute (CIP and IBU mixed) backgrounds. When individually loaded carbons were regenerated, the results indicated that both CIP and IBU exhibited high regeneration efficiencies approaching 100%; however, their regeneration trends and capacities varied significantly. For instance, for CIP alone (Fig. 7a), the regeneration efficiency increased gradually over time, reaching approximately 100% after 180 min. Additionally, the regeneration capacity (*q*_t_) followed the similar trend, increasing progressively up to 150 mg/g within 180 min, indicating CIP’s gradual release and degradation, which was evidenced by the rising fluoride concentration with regeneration time (Fig. S1). In contrast, IBU alone (Fig. 7b) demonstrated much faster regeneration behavior over time, achieving high efficiency and maximum regeneration capacity (150 mg/g) within only 60 min. The slower regeneration of CIP compared to IBU can be explained by its functional groups, such as piperazine ring and carboxylic acid (Cuprys et al. 2018), which enable it to form strong interactions with the carbon surface through various binding modes, including electrostatic (π-π and ionic) interaction, hydrogen bonding, and hydrophobic interaction (Akbari et al. 2025; Yasmin et al. 2024). Furthermore, Onwuka et al. (2025) reported that IBU demonstrates greater electrophilicity than CIP, which enhances its ability to modify its electronic density (Onwuka et al. 2025). Consequently, IBU will be more susceptible toward electrophilic attacks by radicals such as ^•^OH, leading to faster regeneration/degradation than CIP.

On the other hand, in the mixed system (Fig. 7c), both CIP and IBU showed slightly lower regeneration performance compared to their individual cases. For example, CIP in the mixed system required 360 min to achieve complete degradation compared to 180 min in the individual form, while IBU required 180 min compared to 60 min in the individual case. The slower degradation rate for CIP may be attributed to its competition with IBU for the reactive species (e.g., •OH) generated at the BDD anode surface. Since IBU is more electrophilic and exhibits higher reactivity toward •OH through electrophilic substitution, its degradation occurred faster than that of CIP. Furthermore, although CIP in the mixed matrix followed a similar increasing trend to its individual form, both regeneration efficiency and capacity were notably lower. Specifically, regeneration efficiency was suppressed by ~ 65%, and regeneration capacity was reduced by ~ 70% during the early stages of the process, indicating that the recovery of CIP occurred more slowly in the mixture system. Likewise, IBU also exhibited a slightly lower (~ 25%) regeneration efficiency performance, but it was less affected than CIP. As discussed previously, in Section "Adsorption mechanism of CIP and IBU on M-PAC in single solute system", this could be due to differences in the electrostatic or hydrophobic-hydrophobic interactions between CIP and the M-PAC compared to IBU, or possibly due to competitive desorption, where CIP might have had a higher affinity for the M-PAC surface. Therefore, the electro-regeneration process may not be equally effective for all compounds in a mixture, and the effectiveness might depend on the specific properties of the compounds involved, such as molecular size, charge, or polarity, which could influence their retention on the M-PAC. Moreover, the calculated energy consumption (EC) for the electro-regeneration of M-PAC was evaluated for both single-solute and multi-solute systems. Based on the calculations for spent M-PAC, the EC values were 0.05 kWh/g and 0.02 kWh/g for CIP alone and IBU alone, respectively, whereas in the multi-solute system, the EC for CIP and IBU was 0.11 kWh/g and 0.05 kWh/g, respectively. These energy consumption values are lower than those reported for conventional regeneration methods. For instance, thermal and microwave regeneration have been reported to require >900 kWh/kg (Gagliano et al. 2021; Ersan et al. 2023c), highlighting electro-regeneration as a potentially energy-efficient and cost-effective strategy for regenerating spent adsorbents.

Future work could explore the regeneration of M-PAC in more complex real-world mixtures, providing insights into its potential application for broader environmental remediation strategies. All electro-regeneration experiments reported in this study were conducted over a single adsorption-regeneration cycle to evaluate regeneration feasibility, kinetics, and compound-specific behavior, while multi-cycle reusability of M-PAC is identified as an important subject for future investigation.

## Conclusion

In this study, adsorption and ex-situ electro-regeneration of pharmaceutical contaminants (CIP and IBU) on magnetically enhanced iron-modified M-PAC were investigated. Single-solute (DI) and multi-solute (WW) backgrounds were compared side by side and simultaneously. The overall summary of key findings is as follows:

- The settling test results showed that Fe modification improved the settling properties of PAC. Thus, M-PAC can be easily separated without the need for complex filtration or coagulation/flocculation, making it a promising adsorbent when ex-situ regeneration is planned.
- While CIP uptake was higher than IBU by PAC (*K*_F_ = 102 for CIP, *K*_F_ = 63.2 for IBU), their adsorption efficiency on M-PAC was similar (*K*_F_ = 40.1 for CIP, *K*_F_ = 47.7 for IBU) in DI background. This suggests the adsorption of both pharmaceuticals on M-PAC was not influenced by molecular size and properties of pharmaceuticals.
- Simultaneous adsorption experiments showed that the adsorption of CIP (log*K*_ow_ = 0.42) on near-neutrally charged M-PAC decreased due to stronger competition from the more hydrophobic IBU molecule (log*K*_ow_ = 3.5). Furthermore, as the equilibrium concentrations of both pharmaceuticals (particularly IBU) increased, their competitive effect on M-PAC adsorption diminished.
- In the presence of WW background, containing effluent organic matter and inorganics, adsorption capacity of both CIP (*K*_F_ = 28.7) and IBU (*K*_F_ = 15.47) on M-PAC decreased by 28% and 67%, respectively.
- The ex-situ electro-regeneration of M-PAC was effective for both CIP and IBU, with efficiencies reaching 100% in single-solute system.
- For single-solute system, IBU regenerated much faster (60 min) than CIP (180 min) due to its higher reactivity with reactive species through electrophilic substitution.
- In multi-solute system, regeneration was diminished for both CIP (360 min) and IBU (180 min) compared to their individual regeneration efficiencies due to competition for active sites and reactive species.
- The low energy consumption (< 0.2 kWh/g) highlights electro-regeneration as a sustainable and energy-efficient alternative to conventional regeneration methods, allowing in situ reuse of spent adsorbents while minimizing waste generation and reducing reliance on landfilling.
- Future research is needed to examine reusability, capacity recovery, and potential performance of electro-regeneration under multi-cycles (≥ 5 cycles).

## Supplementary Information

Below is the link to the electronic supplementary material.

ESM 1 (DOCX 868 KB)

## Data Availability

All data supporting this manuscript are available upon request.

## References

[CR1] Abdulrahman HH, Sdiq AFH, Ismail HK, Omer RA, Alesary HF, Kareem AA, Barton S (2025) Polymer nanocomposite adsorbents for the removal of pharmaceutical formulations the aquatic environment: a review. Water Air Soil Pollut 236:584. 10.1007/s11270-025-08240-3

[CR2] Akbari A, Peighambardoust SJ, Kazemian H (2025) Comparative study on the impact of physicochemical characteristics of the activated carbons derived from biochar/hydrochar on the adsorption performances. Environ Res 270:121022. 10.1016/j.envres.2025.121022 39914715 10.1016/j.envres.2025.121022

[CR3] Almanassra IW, Kochkodan V, Ponnusamy G, McKay G, Atieh MA, Al-Ansari T (2020) Carbide derived carbon (CDC) as novel adsorbent for ibuprofen removal from synthetic water and treated sewage effluent. J Environ Health Sci Eng 18:1375–1390. 10.1007/s40201-020-00554-0 33312649 10.1007/s40201-020-00554-0 PMC7721931

[CR4] aus der Beek T, Weber F-A, Bergmann A, Hickmann S, Ebert I, Hein A, Küster A (2015) Pharmaceuticals in the environment—global occurrences and perspectives. Environ Toxicol Chem 35:823–835. 10.1002/etc.3339 10.1002/etc.3339 26666847

[CR5] Bakkaloglu S, Ersan MS, Karanfil T, Apul OG (2021) Effect of superfine pulverization of powdered activated carbon on adsorption of carbamazepine in natural source waters. Sci Total Environ 793:148473. 10.1016/j.scitotenv.2021.148473 34328993 10.1016/j.scitotenv.2021.148473

[CR6] Brasquet C, Le Cloirec P (1997) Adsorption onto activated carbon fibers: application to water and air treatments. Carbon 35:1307–1313. 10.1016/S0008-6223(97)00079-1

[CR7] Chen C, Apul OG, Karanfil T (2017) Removal of bromide from surface waters using silver impregnated activated carbon. Water Res 113:223–230. 10.1016/j.watres.2017.01.019 28226281 10.1016/j.watres.2017.01.019

[CR8] Cuprys A, Pulicharla R, Lecka J, Brar SK, Drogui P, Surampalli RY (2018) Ciprofloxacin-metal complexes –stability and toxicity tests in the presence of humic substances. Chemosphere 202:549–559. 10.1016/j.chemosphere.2018.03.117 29587236 10.1016/j.chemosphere.2018.03.117

[CR9] Ding L, Snoeyink VL, Mariñas BJ, Yue Z, Economy J (2008) Effects of powdered activated carbon pore size distribution on the competitive adsorption of aqueous atrazine and natural organic matter. Environ Sci Technol 42:1227–1231. 10.1021/es0710555 18351097 10.1021/es0710555

[CR10] Dittmann D, Saal L, Zietzschmann F, Mai M, Altmann K, Al D, Pia S, Ruhl AS, Jekel M, Braun U (2022) Characterization of activated carbons for water treatment using TGA-FTIR for analysis of oxygen-containing functional groups. Appl Water Sci 12:203. 10.1007/s13201-022-01723-2

[CR11] Ersan G, Cerr´on-Calle GA, Ersan MS, Garcia-Segura S (2023a) Opportunities for in situ electro-regeneration of organic contaminant-laden carbonaceous adsorbents. Water Res 232:119718. 10.1016/j.watres.2023.119718 36774755 10.1016/j.watres.2023.119718

[CR12] Ersan G, dos Santos AJ, Lanza MRV, Perreault F, Garcia-Segura S (2023b) Enhancing the selective ciprofloxacin adsorption in urine matrices through the metal-doping of carbon sorbents. J Environ Manage 348:119298. 10.1016/j.jenvman.2023.119298 37839202 10.1016/j.jenvman.2023.119298

[CR13] Ersan G, Ersan MS, Perreault F, Garcia-Segura S (2023c) Enabling in situ electro-regeneration systems for PFOA-laden spent activated carbon adsorbents reuse. J Environ Chem Eng 11(6):111369. 10.1016/j.jece.2023.111369

[CR14] Ersan G, Gaber MS, Perreault F, Garcia-Segura S (2024) Comparative study on electro-regeneration of antibiotic-laden activated carbons in reverse osmosis concentrate. Water Res 255:121528. 10.1016/j.watres.2024.121528 38555781 10.1016/j.watres.2024.121528

[CR15] Fanourakis SK, Peña-Bahamonde J, Bandara PC, Rodrigues DF (2020) Nano-based adsorbent and photocatalyst use for pharmaceutical contaminant removal during indirect potable water reuse. NPJ Clean Water 3:1. 10.1038/s41545-019-0048-8

[CR16] Gaber MS, Salah BA, Kandil AT (2023) Adsorption of yttrium (III), neodymium (III), gadolinium (III), samarium (III), and lutetium (III) ions using 8-hydroxyquinoline intercalated bentonite. Desalin Water Treat 282:127–138. 10.5004/dwt.2023.29153

[CR17] Gagliano E, Falciglia PP, Zaker Y, Karanfil T, Roccaro P (2021) Microwave regeneration of granular activated carbon saturated with PFAS. Water Res 198:117121. 10.1016/j.watres.2021.117121 33910144 10.1016/j.watres.2021.117121

[CR18] Gaudino S, Galas C, Belli M, Barbizzi S, de Zorzi P, Jaćimović R, Sansone U (2007) The role of different soil sample digestion methods on trace elements analysis: a comparison of ICP-MS and INAA measurement results. Accred Qual Assur 12:84–93. 10.1007/s00769-006-0238-1

[CR19] Gezahegn T, Tegegne B, Zewge F, Chandravanshi BS (2019) Salting-out assisted liquid–liquid extraction for the determination of ciprofloxacin residues in water samples by high performance liquid chromatography–diode array detector. BMC Chem 13:28. 10.1186/s13065-019-0543-5 31384776 10.1186/s13065-019-0543-5 PMC6661818

[CR20] Guillossou R, Le Roux J, Mailler R, Pereira-Derome CS, Varrault G, Bressy A, Vulliet E, Morlay C, Nauleau F, Rocher V, Gasperi J (2020) Influence of dissolved organic matter on the removal of 12 organic micropollutants from wastewater effluent by powdered activated carbon adsorption. Water Res 172:115487. 10.1016/j.watres.2020.115487 31962270 10.1016/j.watres.2020.115487

[CR21] Hama Aziz KH, Mustafa FS, Karim MAH, Hama S (2025) Pharmaceutical pollution in the aquatic environment: advanced oxidation processes as efficient treatment approaches: a review. Mater Adv 6:3433–3454. 10.1039/D4MA01122H

[CR22] Jafari AJ, Kakavandi B, Kalantary RR, Gharibi H, Asadi A, Azari A, Babaei AA, Takdastan A (2016) Application of mesoporous magnetic carbon composite for reactive dyes removal: process optimization using response surface methodology. Korean J Chem Eng 33:2878–2890. 10.1007/s11814-016-0155-x

[CR23] Janecko N, Pokludova L, Blahova J, Svobodova Z, Literak I (2016) Implications of fluoroquinolone contamination for the aquatic environment—a review. Environ Toxicol Chem 35:2647–2656. 10.1002/etc.3552 27392330 10.1002/etc.3552

[CR24] Jothinathan L, Cai QQ, Ong SL, Hu JY (2022) Fe-Mn doped powdered activated carbon pellet as ozone catalyst for cost-effective phenolic wastewater treatment: mechanism studies and phenol by-products elimination. J Hazard Mater 424:127483. 10.1016/j.jhazmat.2021.127483 34673392 10.1016/j.jhazmat.2021.127483

[CR25] Kårelid V, Larsson G, Björlenius B (2017) Pilot-scale removal of pharmaceuticals in municipal wastewater: comparison of granular and powdered activated carbon treatment at three wastewater treatment plants. J Environ Manage 193:491–502. 10.1016/j.jenvman.2017.02.042 28256364 10.1016/j.jenvman.2017.02.042

[CR26] Krahnstöver T, Wintgens T (2018) Separating powdered activated carbon (PAC) from wastewater – technical process options and assessment of removal efficiency. J Environ Chem Eng 6:5744–5762. 10.1016/j.jece.2018.09.001

[CR27] Le TP, Luong HVT, Nguyen HN, Pham TKT, Le TLT, Tran TBQ, Ngo TNM (2024) Insight into adsorption-desorption of methylene blue in water using zeolite NaY: kinetic, isotherm and thermodynamic approaches. Results in Surfaces and Interfaces 16:100281. 10.1016/j.rsurfi.2024.100281

[CR28] Li M, Fu S, Han Y, Zheng J, Wang C, Xu X, Zhu L (2024) Synergistic removal of carbon and phosphorus by modified carbon-based magnetic materials. Chem Eng J 491:151244. 10.1016/j.cej.2024.151244

[CR29] Ling AL, Stegner T, Thompson M, Viswanathan S, Pinkard BR, Wolohan KM, Ellis A (2025) Spent media management pathways for PFAS treatment applications. Water Environ Res 97(7):e70130. 10.1002/wer.70130 40660761 10.1002/wer.70130 PMC12260480

[CR30] Luján-Facundo MJ, Iborra-Clar MI, Mendoza-Roca JA, Alcaina-Miranda MI (2019) Pharmaceutical compounds removal by adsorption with commercial and reused carbon coming from a drinking water treatment plant. J Clean Prod 238:117866. 10.1016/j.jclepro.2019.117866

[CR31] Margot J, Kienle C, Magnet A, Weil M, Rossi L, Alencastro LFd, Abegglen C, Thonney D, Chevre N, Scharer M, Barry DA (2013) Treatment of micropollutants in municipal wastewater: ozone or powdered activated carbon? Sci Total Environ 461:480–498. 10.1016/j.scitotenv.2013.05.034 23751332 10.1016/j.scitotenv.2013.05.034

[CR32] Márquez P, Benítez A, Chica AF, Martín MA, Caballero A (2022) Evaluating the thermal regeneration process of massively generated granular activated carbons for their reuse in wastewater treatments plants. J Clean Prod 366:132685. 10.1016/j.jclepro.2022.132685

[CR33] Morsch P, Möhlendick L, Süsser M, Nirschl H (2021) Elimination of micropollutants from municipal wastewater by adsorption on powdered activated carbon and separation by innovative precoat filtration. Sep Purif Technol 277:119444. 10.1016/j.seppur.2021.119444

[CR34] Oliveira LCA, Rios RV, Fabris JD, Garg V, Sapag K, Lago RM (2002) Activated carbon/iron oxide magnetic composites for the adsorption of contaminants in water. Carbon N Y 40:2177–2183. 10.1016/S0008-6223(02)00076-3

[CR35] Onwuka KE, Ahuchaogu AA, Atasie OC, Oguike VC, Nwosu AC, Nwosu NB, Nwagba JO (2025) Comparative adsorption of ibuprofen and ciprofloxacin from aqueous solution using natural bentonite clay: experimental and DFT study. Acta Tech Jaurinensis 18:138–155. 10.14513/actatechjaur.00774

[CR36] Parniske J, Al-Asad HA, Qian J, Morck T (2024) Modelling competitive adsorption of organic micropollutants onto powdered activated carbon in continuous stirred tank reactors for advanced wastewater treatment. Water Res 258:121806. 10.1016/j.watres.2024.121806 38796911 10.1016/j.watres.2024.121806

[CR37] Patel A, Panter GH, Trollope HT, Glennon YC, Owen SF, Sumpter JP, Rand-Weaver M (2016) Testing the “read-across hypothesis” by investigating the effects of ibuprofen on fish. Chemosphere 163:592–600. 10.1016/j.chemosphere.2016.08.041 27572306 10.1016/j.chemosphere.2016.08.041 PMC5034852

[CR38] Shearer L, Pap S, Gibb SW (2022) Removal of pharmaceuticals from wastewater: a review of adsorptive approaches, modelling and mechanisms for metformin and macrolides. J Environ Chem Eng 10:108106. 10.1016/j.jece.2022.108106

[CR39] Shon HK, Vigneswaran S, Kandasamy J, Cho J (2007) Characteristics of effluent organic matter in wastewater. Eolss Publisher Co., Ltd, Oxford, UK

[CR40] Siriwardena DP, James R, Dasu K, Thorn J, Iery RD, Pala F, Schumitz D, Eastwood S, Burkitt N (2021) Regeneration of per-and polyfluoroalkyl substance-laden granular activated carbon using a solvent based technology. J Environ Manage 289:112439. 10.1016/j.jenvman.2021.112439 33819657 10.1016/j.jenvman.2021.112439

[CR41] Stumm W, Morgan JJ (1996) Aquatic chemistry: chemical equilibria and rates in natural, 3rd edn. Environmental Science and Technology, New York

[CR42] Thai V-A, Dang VD, Thuy NT, Pandit B, Vo TKQ, Khedulkar AP (2023) Fluoroquinolones: fate, effects on the environment and selected removal methods. J Clean Prod 418:137762. 10.1016/j.jclepro.2023.137762

[CR43] Uddin AH, Khalid RS, Alaama M, Abdualkader AM, Kasmuri A, Abbas SA (2016) Comparative study of three digestion methods for elemental analysis in traditional medicine products using atomic absorption spectrometry. J Anal Sci Technol 7:6. 10.1186/s40543-016-0085-6

[CR44] Vazquez-Roig P, Andreu V, Onghena M, Blasco C, Picó Y (2011) Assessment of the occurrence and distribution of pharmaceuticals in a Mediterranean wetland (L’Albufera, Valencia, Spain) by LC-MS/MS. Anal Bioanal Chem 400:1287–1301. 10.1007/s00216-011-4826-5 21416164 10.1007/s00216-011-4826-5

[CR45] Watkinson AJ, Murby EJ, Kolpin DW, Costanzo SD (2009) The occurrence of antibiotics in an urban watershed: from wastewater to drinking water. Sci Total Environ 407:2711–2723. 10.1016/j.scitotenv.2008.11.059 19138787 10.1016/j.scitotenv.2008.11.059

[CR46] Woermann M, Zimmermann S, Sures B (2020) Is micropollutant-loaded powdered activated carbon from a wastewater treatment plant toxic to the bivalve *Corbicula* sp.? Environ Sci Eur 32:151. 10.1186/s12302-020-00430-6 10.1016/j.scitotenv.2020.141104 32763603

[CR47] Yasmin S, Azam MG, Hossain MS, Akhtar U, Kabir M (2024) Efficient removal of ciprofloxacin from aqueous solution using Zn–C battery derived graphene oxide enhanced by hydrogen bonding, electrostatic and π-π interaction. Heliyon 10:e33317. 10.1016/j.heliyon.2024.e33317 39022076 10.1016/j.heliyon.2024.e33317 PMC11253669

[CR48] Zhou Q, Yu Z, Ma Y (2019) Review on the application of magnetic flocculation technology in water treatment. IOP Conf Ser Earth Environ Sci 295:042107. 10.1088/1755-1315/295/4/042107

