# Supplementary material for: Pharmaceutical adsorption and ex-situ electro-regeneration performance of magnetically modified activated carbon
Source: Environ Sci Pollut Res Int. 2026 Mar 25;33(12):5716–30. doi: 10.1007/s11356-026-37655-6 (PMC13091856; doi:10.1007/s11356-026-37655-6)
Supplement: Supplementary file 1 — (DOCX 868 KB) [file 11356_2026_37655_MOESM1_ESM.docx]

SUPPORTING INFORMATION

*For*

### **Pharmaceutical Adsorption and Ex-situ Electro-Regeneration Performance of Magnetically Modified Activated Carbon**

Faisal Ahmed^1ǂ^, Mohamed S. Gaber^1^, Gamze Ersan^1ǂ^, Sergi Garcia-Segura^2^, Mahmut S. Ersan^1,*^

*^1^Department of Civil Engineering, University of North Dakota, Grand Forks, ND 58202-8115, USA*

*^2^School of Sustainable Engineering and The Built Environment, Arizona State University, Tempe, AZ 85287-5306, USA*

*Submitted to*

*Environmental Science and Pollution Research*

*March 12^th^, 2026*

ǂ These authors contributed equally to this work

*Corresponding author: [mahmut.ersan@und.edu](mailto:mahmut.ersan@und.edu) (Mahmut S. Ersan)

**Table S1.** Physicochemical characteristics of selected pharmaceutical chemicals

| **Pharmaceuticals** | **Physicochemical characteristics** | |
| --- | --- | --- |
| **Ciprofloxacin (CIP)** | **Chemical formula** | C_17_H_18_FN_3_O_3_ |
|  | **Molar weight (g mol^-1^)** | 331.4 |
|  | **Solubility in water (mg L^-1^)** | 120 (298K) |
|  | **p*K*_a_** | 6.1- 8.7^a^ |
|  | **log *K*_ow_** | 0.42 (298K) |
|  | **Molecular size (Å)** | 8 x 11 x 15 |
|  | **3D Molecular structure** | 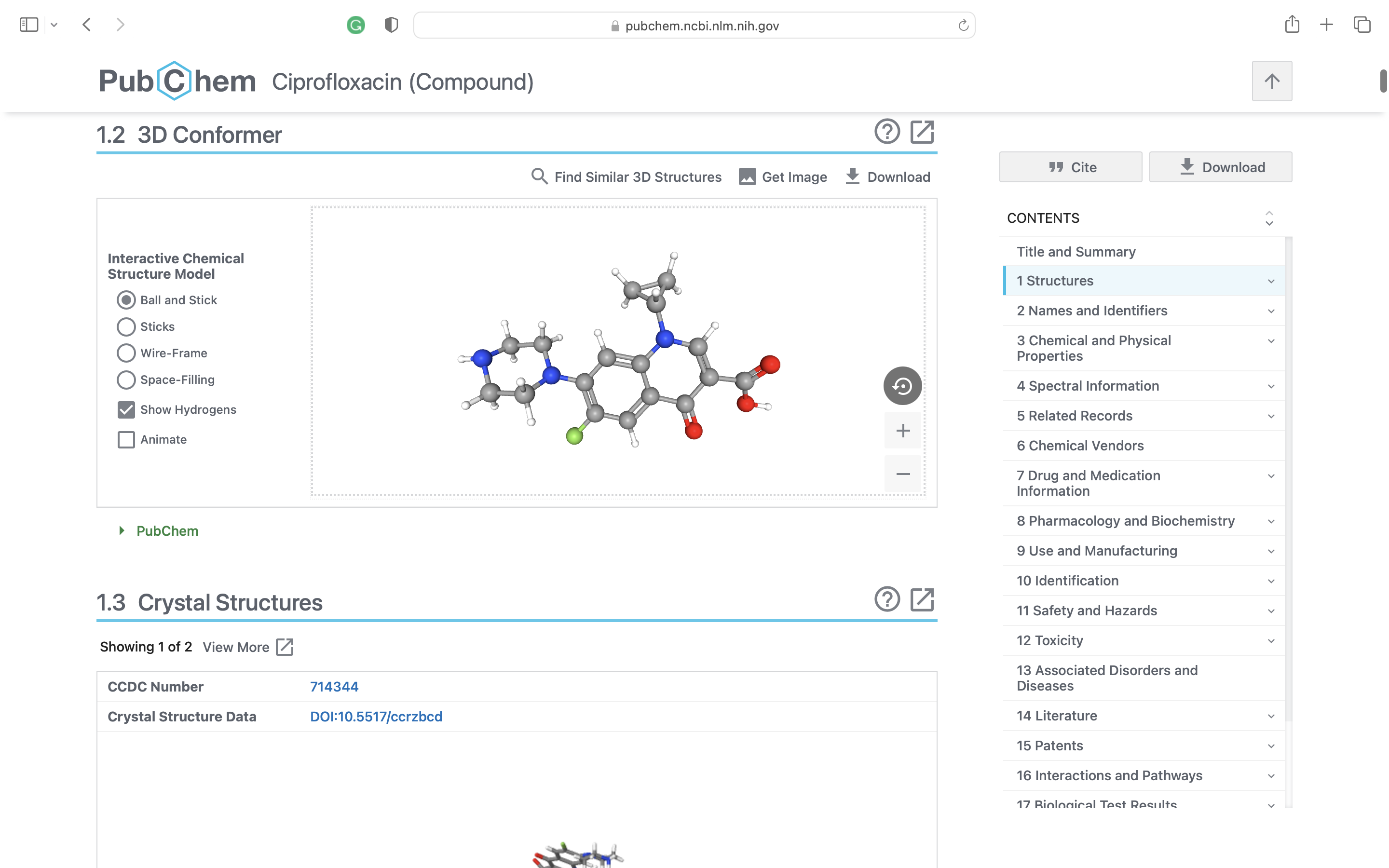 |
| **Ibuprofen (IBU)** | **Chemical formula** | C_13_H_18_O_2_ |
|  | **Molar weight (g mol^-1^)** | 206.3 |
|  | **Solubility in water (mg L^-1^)** | 0.021 (273K) |
|  | **p*K*_a_** | 4.52^b^ |
|  | **log *K*_ow_** | 3.5 |
|  | **Molecular size (Å)** | 10.3 x 5.2 x 4.3 |
|  | **3D Molecular structure** | 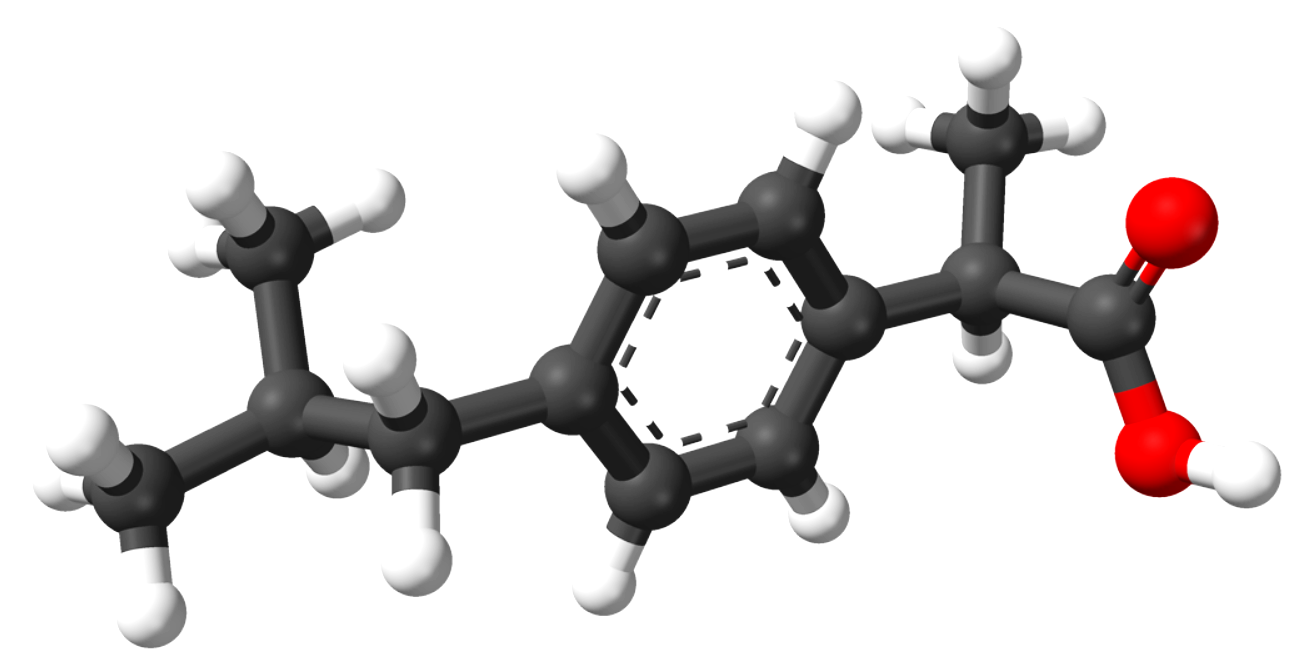 |

References: ^a^ (Gu and Karthikeyan 2005), ^b^ (Ràfols et al. 1997).

**Table S2.** Selected Adsorption Isotherm Models

| **Model** | **Abbreviation** | **Equation** | **Terms** |
| --- | --- | --- | --- |
| Freundlich | FM | $q_{e}=K_{F}C_{e}^{n}$ | K_F_ [(mg/g)/(L/μg)_n_]: adsorption affinity coefficient  n: nonlinear index |
| Langmuir | LM | $q_{e}=\frac{q_{m}K_{L}C_{e}}{1+K_{L}C_{e}}$ | K_L_[(L/μg)]: adsorption affinity coefficient |

^*^ q_e_ [mg/g]: equilibrium concentration of solute in adsorbent, q_m_ [mg/g]: maximum adsorption capacity, C_e_: [μg/L] equilibrium concentration of solute in solution.

**Table S3-a.** Settling test Pristine PAC and magnetic Fe-doped PACs

| **Pristine PAC** | | | |
| --- | --- | --- | --- |
| **Time (min)** | **Absorbance** | **Time (min)** | **Absorbance** |
| 0 | 0.862 | 24 | 0.331 |
| 1 | 0.821 | 25 | 0.327 |
| 2 | 0.592 | 26 | 0.32 |
| 3 | 0.563 | 27 | 0.318 |
| 4 | 0.539 | 28 | 0.308 |
| 5 | 0.516 | 29 | 0.297 |
| 6 | 0.498 | 30 | 0.285 |
| 7 | 0.481 | 31 | 0.274 |
| 8 | 0.469 | 32 | 0.261 |
| 9 | 0.458 | 33 | 0.251 |
| 10 | 0.442 | 34 | 0.239 |
| 11 | 0.432 | 35 | 0.229 |
| 12 | 0.422 | 36 | 0.211 |
| 13 | 0.412 | 37 | 0.199 |
| 14 | 0.403 | 38 | 0.182 |
| 15 | 0.395 | 39 | 0.166 |
| 16 | 0.388 | 40 | 0.151 |
| 17 | 0.376 | 41 | 0.131 |
| 18 | 0.367 | 42 | 0.089 |
| 19 | 0.361 | 43 | 0.015 |
| 20 | 0.352 | 44 | 0.009 |
| 21 | 0.348 | 45 | 0.004 |
| 22 | 0.34 |  |  |
| 23 | 0.339 |  |  |

**Table S3-b.** Settling test Magnetic Fe-doped PACs

| **Magnetic PAC** | | | |
| --- | --- | --- | --- |
| **Time (min)** | **Absorbance** | **Time (min)** | **Absorbance** |
| 0 | 0.36 | 15 | 0.152 |
| 1 | 0.291 | 16 | 0.149 |
| 2 | 0.267 | 17 | 0.146 |
| 3 | 0.251 | 18 | 0.143 |
| 4 | 0.238 | 19 | 0.139 |
| 5 | 0.218 | 20 | 0.136 |
| 6 | 0.202 | 21 | 0.133 |
| 7 | 0.197 | 22 | 0.112 |
| 8 | 0.19 | 23 | 0.097 |
| 9 | 0.184 | 24 | 0.091 |
| 10 | 0.176 | 25 | 0.011 |
| 11 | 0.172 | 26 | 0.009 |
| 12 | 0.167 | 27 | 0.004 |
| 13 | 0.161 |  |  |
| 14 | 0.156 |  |  |


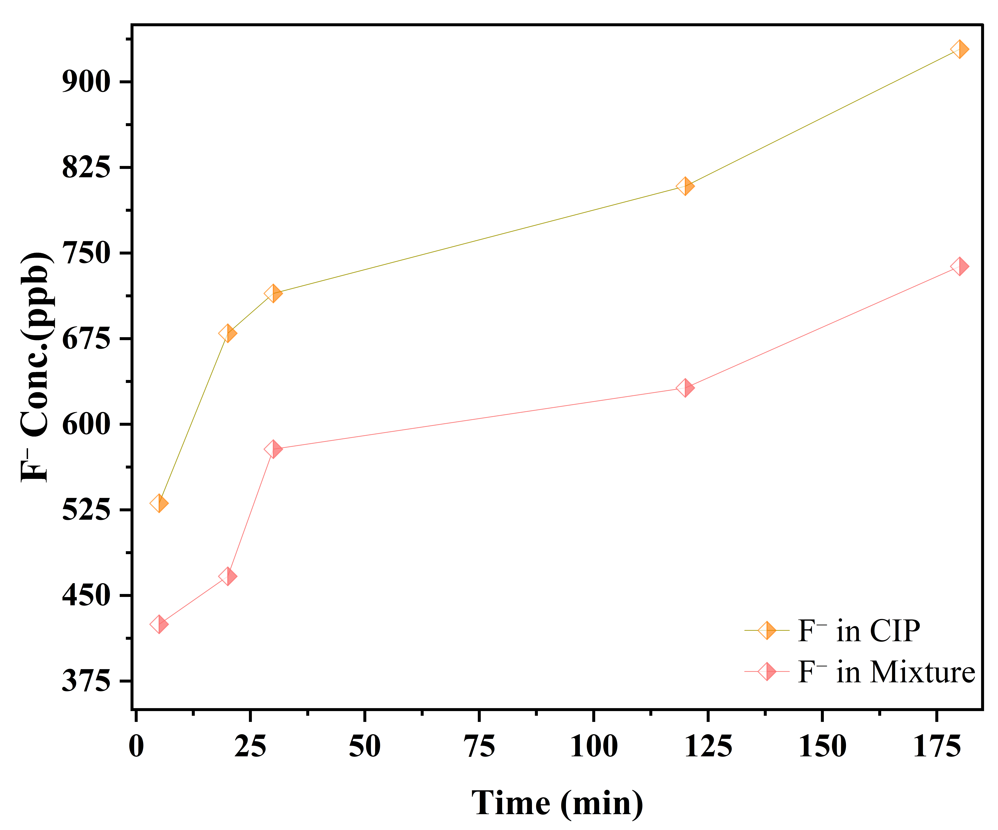


**Figure S1.** Fluoride (F^-^) concentration in CIP and mixture of CIP and IBU during regeneration

**References**

Gu C, Karthikeyan KG (2005) Sorption of the Antimicrobial Ciprofloxacin to Aluminum and Iron Hydrous Oxides. Environ Sci Technol 39:9166–9173. https://doi.org/10.1021/es051109f

Ràfols C, Rosés M, Bosch E (1997) A comparison between different approaches to estimate the aqueous pKa values of several non-steroidal anti-inflammatory drugs. Anal Chim Acta 338:127–134. https://doi.org/10.1016/S0003-2670(96)00496-5
